# Prevalence of alcohol and other drug detections in non‐transport injury events

**DOI:** 10.1111/1742-6723.14312

**Published:** 2023-09-17

**Authors:** Georgina Lau, Biswadev Mitra, Belinda J Gabbe, Paul M Dietze, Sandra Reeder, Peter A Cameron, De Villiers Smit, Hans G Schneider, Evan Symons, Christine Koolstra, Cara Stewart, Ben Beck

**Affiliations:** ^1^ School of Public Health and Preventive Medicine Monash University Melbourne Victoria Australia; ^2^ Emergency and Trauma Centre The Alfred Hospital Melbourne Victoria Australia; ^3^ Health Data Research UK, Swansea University Medical School Swansea University Swansea UK; ^4^ Disease Elimination Program, Burnet Institute Melbourne Victoria Australia; ^5^ National Drug Research Institute Curtin University Perth Western Australia Australia; ^6^ Central Clinical School Monash University Melbourne Victoria Australia; ^7^ National Trauma Research Institute The Alfred Hospital Melbourne Victoria Australia; ^8^ Department of Pathology The Alfred Hospital Melbourne Victoria Australia; ^9^ Alfred Mental and Addiction Health The Alfred Hospital Melbourne Victoria Australia

**Keywords:** alcoholic intoxication, blood alcohol content, illicit drug, substance use detection, substance‐related disorder, wounds and injury

## Abstract

**Objective:**

To measure the prevalence of alcohol and/or other drug (AOD) detections in suspected major trauma patients with non‐transport injuries who presented to an adult major trauma centre.

**Methods:**

This registry‐based cohort study examined the prevalence of AOD detections in patients aged ≥18 years who: (i) sustained non‐transport injuries; and (ii) met predefined trauma call‐out criteria and were therefore managed by an interdisciplinary trauma team between 1 July 2021 and 31 December 2022. Prevalence was measured using routine in‐hospital blood alcohol and urine drug screens.

**Results:**

A total of 1469 cases met the inclusion criteria. Of cases with a valid blood test (*n* = 1248, 85.0%), alcohol was detected in 313 (25.1%) patients. Of the 733 (49.9%) cases with urine drug screen results, cannabinoids were most commonly detected (*n* = 103, 14.1%), followed by benzodiazepines (*n* = 98, 13.4%), amphetamine‐type substances (*n* = 80, 10.9%), opioids (*n* = 28, 3.8%) and cocaine (*n* = 17, 2.3%). Alcohol and/or at least one other drug was detected in 37.4% (*n* = 472) of cases with either a blood alcohol or urine drug test completed (*n* = 1263, 86.0%). Multiple substances were detected in 16.6% (*n* = 119) of cases with both blood alcohol and urine drug screens (*n* = 718, 48.9%). Detections were prevalent in cases of interpersonal violence (*n* = 123/179, 68.7%) and intentional self‐harm (*n* = 50/106, 47.2%), and in those occurring on Friday and Saturday nights (*n* = 118/191, 61.8%).

**Conclusion:**

AOD detections were common in trauma patients with non‐transport injury causes. Population‐level surveillance is needed to inform prevention strategies that address AOD use as a significant risk factor for serious injury.

## Graphical abstract

Based on the results of routine blood alcohol and urine drug screens, alcohol and/or other drugs were detected in 37% of trauma patients with non‐transport injury causes. Alcohol and/or other drug detections were particularly common in cases of intentional self‐harm (47%) and interpersonal violence (69%). Alcohol was the most commonly detected substance (25%), followed by cannabinoids (14%), benzodiazepines (13%), amphetamine‐type substances (11%), opioids (4%) and cocaine (2%).

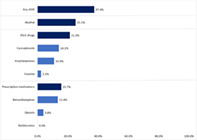

**Key findings**

- AODs were detected in 37% of non‐transport injury cases where AOD testing was completed. AOD detections were particularly common in cases of intentional self‐harm (47%) and interpersonal violence (69%).
- Of tested patients, alcohol was the most commonly detected substance (25%), followed by cannabinoids (14%), benzodiazepines (13%), amphetamine‐type substances (11%), opioids (4%) and cocaine (2%).
- Population‐level surveillance of AOD‐related injury events is needed to inform prevention approaches that address AOD use as a risk factor for all causes of injury.

## Introduction

Alcohol and/or other drug (AOD) use is a key modifiable risk factor for serious injury. Research and prevention efforts to date have largely focused on drink and drug driving, leading to decreases in AOD‐related motor vehicle collisions in Australia and internationally.^1^ Recent Australian research reported that AODs were detected in 38% of injured drivers, including alcohol detections in 16% of the sample.^2^ Meanwhile, knowledge on the prevalence and role of AODs in non‐transport injury events such as falls, violence and intentional self‐harm remains limited. This is despite non‐transport injury causes comprising 66% of major trauma injuries.^3^

Systematic reviews indicate that AODs are detected in 37% of fall‐related injuries and up to 77% of violence‐related injuries.^4^, ^5^ However, most studies in these systematic reviews were from the United States and prevalence estimates can vary substantially between countries.^6^ Contemporaneous Australian data on the prevalence of AODs in non‐transport injury events are lacking and existing estimates may not be relevant in Australia, where AOD regulations differ (e.g. different minimum drinking ages, recreational cannabis regulation). Together with varying AOD use cultures, these differences in regulation and availability may influence AOD use behaviours and associated harms.^7^

Robust prevalence estimates of AOD detections in injury events are important for the development and implementation of evidence‐informed prevention strategies.^8^ In the present study, we aim to provide recent estimates of the prevalence of AOD detections in cases of non‐transport injury at a major trauma centre in Victoria, Australia.

## Methods

Patients who presented to the Emergency and Trauma Centre at The Alfred Hospital between 1 July 2021 and 31 December 2022 were examined. Patients were included if they (i) were aged ≥18 years; and (ii) met predefined trauma call‐out criteria and were therefore managed by an interdisciplinary trauma team (see Cameron *et al.* ^9^ for detailed trauma call‐out criteria). At The Alfred Hospital, there are multiple activation protocols for a trauma call‐out. These activation protocols use a variety of physiological, mechanistic and patient characteristics to predict injury severity.^9^ The Alfred Hospital is one of two designated adult major trauma centres in Victoria and has routinely ordered blood alcohol and urine drug screens as part of clinical care for all patients meeting trauma call‐out criteria since June 2021. These tests are expected to guide clinical care, such as to assist in the assessment of patients with altered conscious states or to provide objective evidence for discussions and referral to AOD services. The communication of investigation results to patients follows standard clinical processes and is done at clinical discretion. Transport‐related injuries, defined as any injury involving a motorised or human‐powered vehicle that operates on land (e.g. cars, trucks, motorcycles, bicycles, trains, trams, pedestrians struck by a motor vehicle), were excluded. Patients who did not present to hospital directly from the scene of injury were excluded as inter‐hospital transfer times would likely impact on AOD testing and results.

The present study had ethical approval from the Alfred Hospital Human Research and Ethics Committee (124/21). Eligible patients were identified from the Alfred Health Trauma Registry and had medical file reviews completed using a waiver of consent. Data were extracted on patient demographics (age at the time of injury, sex), the injury event (cause, intent, time and date), hospital characteristics (time and date of admission, length of hospital stay, trauma unit admission) and AOD use (home, pre‐hospital and in‐patient medications, AOD test results, time of AOD testing). Patients in the non‐binary sex category were excluded for confidentiality reasons due to low patient numbers. Injury causes were categorised as low falls (<1 m or from standing height), high falls (≥1 m), flames/scalds/contact burns, cutting/piercing objects, being struck by/colliding with an object/person and other (including machinery, animal, drowning and firearm‐related injuries). Intent was classified as unintentional, intentional self‐harm, interpersonal violence and could not be determined. Date and time of injury were used to determine whether the injury event occurred during times associated with high community alcohol consumption, referred to as high alcohol hours (8 pm Friday–6 am Saturday and 8 pm Saturday–6 am Sunday).^10^ The time of blood alcohol and urine drug screens were recorded, enabling calculation of the length of time between injury and testing.

### Alcohol and other drugs

Blood alcohol concentration (BAC) was measured using an alcohol dehydrogenase method and urine drug measurements were obtained by immunoassay. Urine drug screen results are reported in medical records as ‘detected’ or ‘not detected’ for amphetamine‐type substances, barbiturates, benzodiazepines, cannabinoids, cocaine and opioids based on the published cut‐off values of the Australian Standard AS/NZS 4308 (Table S1). Patient BACs are recorded in medical records in mmol/L, with a lower detection limit of 2 mmol/L. Alcohol was considered detected if a BAC ≥2 mmol/L was identified in a patient's blood sample. Patient BAC was converted to g/100 mL for reporting in the present study. Drug detections from urine screens were recoded as ‘not detected’ if medical records indicated that patients were given the drug by paramedics or clinicians at the hospital after the injury event, but before drug testing. Patients with active pre‐injury prescriptions for opioids, benzodiazepines, amphetamine‐type substances, barbiturates or cannabinoids were identified from medical records.

### Data analysis

The sample was described using counts and percentages. Where relevant, mean and standard deviation (SD) or median and interquartile range (IQR) were reported. *χ* ^2^ tests were used to compare patients who had AOD testing with patients who did not. *P*‐values <0.05 were considered significant. Prevalence estimates reflect the proportion of patients with an AOD detection of all patients who had a relevant AOD test completed.

## Results

**Figure 1:**
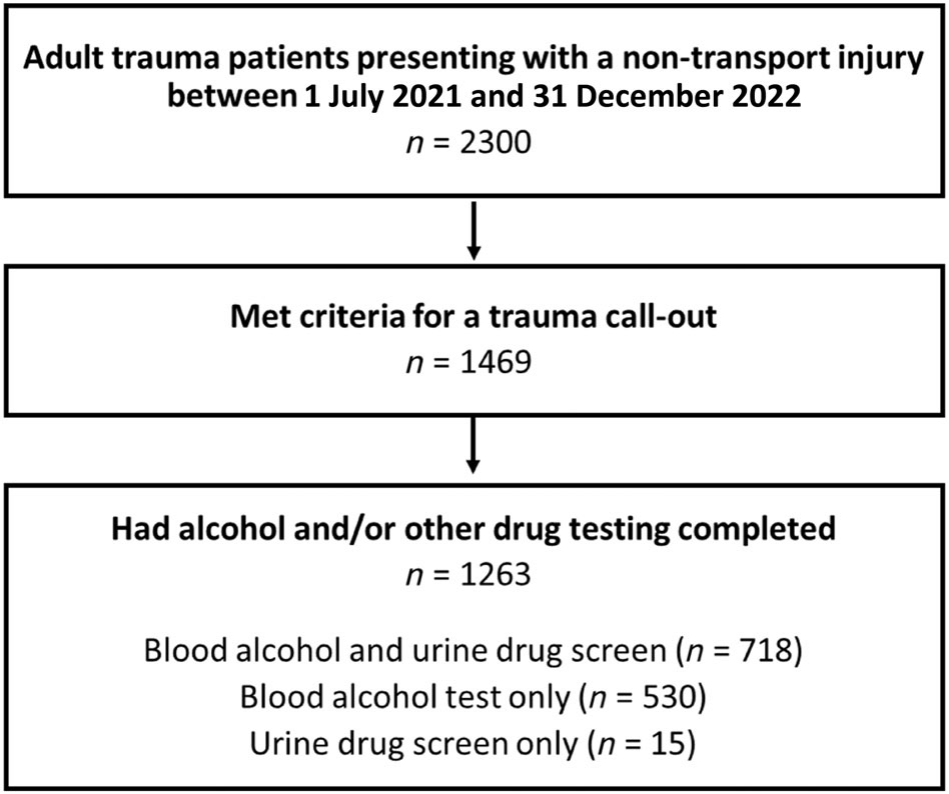
Flowchart summarising alcohol and/or other drug testing in the sample.

A total of 1469 patients were included (Fig. 1). The sample had a mean age of 51.5 years (SD 21.0) and was 72.9% male (*n* = 1071). The most common injury cause was high falls (*n* = 462, 31.4%), followed by low falls (*n* = 354, 24.1%), being struck by/colliding with an object/person (*n* = 196, 13.3%), cutting/piercing objects (*n* = 192, 13.1%) and flames/scalds/contact burns (*n* = 162, 11.0%). Detailed sample characteristics are presented in Table 1.

**TABLE 1:** Comparison of cases with and without blood alcohol tests or urine drug screens completed

	Total sample, *n* (%)	Blood alcohol test, *n* (%)		*P*‐value†	Urine drug screen, *n* (%)		*P*‐value†
		No	Yes		No	Yes	
	1469 (100.0)	221 (15.0)	1248 (85.0)		736 (50.1)	733 (49.9)	
Sex				0.24			**0.04**
Male	1070 (72.9)	154 (69.7)	917 (73.5)		519 (70.5)	552 (75.3)	
Female	398 (27.1)	67 (30.3)	331 (26.5)		217 (29.5)	181 (24.7)	
Age (years)				0.47			0.14
18–24	172 (11.7)	19 (8.6)	153 (12.3)		93 (12.6)	79 (10.8)	
25–34	211 (14.4)	27 (12.2)	184 (14.7)		106 (14.4)	105 (14.3)	
35–44	220 (15.0)	36 (16.3)	184 (14.7)		94 (12.8)	126 (17.2)	
45–54	226 (15.4)	36 (16.3)	190 (15.2)		111 (15.1)	115 (15.7)	
55–64	207 (14.1)	30 (13.6)	177 (14.2)		100 (13.6)	107 (14.6)	
≥65	433 (29.5)	73 (33.0)	360 (28.8)		232 (31.5)	201 (27.4)	
Injury cause				**<0.001**			**<0.001**
Low fall	354 (24.1)	56 (25.3)	298 (23.9)		206 (28.0)	148 (20.2)	
High fall	462 (31.4)	30 (13.6)	432 (34.6)		178 (24.2)	284 (38.7)	
Flames/scalds/contact burns	162 (11.0)	76 (34.4)	86 (6.9)		112 (15.2)	50 (6.8)	
Cutting/piercing objects	192 (13.1)	26 (11.8)	166 (13.3)		98 (13.3)	94 (12.8)	
Struck by/collided with an object/person	196 (13.3)	17 (7.7)	179 (14.3)		93 (12.6)	103 (14.1)	
Other/unknown‡	103 (7.0)	16 (7.2)	87 (7.0)		49 (6.7)	54 (7.4)	
Injury intent				**0.01**			0.44
Unintentional	1094 (74.5)	181 (81.9)	913 (73.2)		556 (75.5)	538 (73.4)	
Intentional self‐harm	121 (8.2)	18 (8.1)	103 (8.3)		56 (7.6)	65 (8.9)	
Interpersonal violence	194 (13.2)	15 (6.8)	179 (14.3)		99 (13.5)	95 (13.0)	
Could not determine	60 (4.1)	7 (3.2)	53 (4.2)		25 (3.4)	35 (4.8)	
High alcohol hours				0.09			0.58
Yes	214 (14.6)	24 (10.9)	190 (15.2)		111 (15.1)	103 (14.1)	
No	1255 (85.4)	197 (89.1)	1058 (84.8)		625 (84.9)	630 (85.9)	
Admitted to trauma				**<0.001**			**<0.001**
Yes	845 (57.5)	87 (39.4)	758 (60.7)		335 (45.5)	510 (69.6)	
No	624 (42.5)	134 (60.6)	490 (39.3)		401 (54.5)	223 (30.4)	
Length of hospital stay (days)				0.09			**<0.001**
≤1	421 (28.7)	74 (33.5)	347 (27.8)		291 (39.5)	130 (17.7)	
>1	1048 (71.3)	147 (66.5)	901 (72.2)		445 (60.5)	603 (82.3)	

† *P*‐values correspond to *χ* ^2^ tests. Significant *P*‐values are shown in bold.

‡ Includes machinery, animal, drowning and firearm‐related injuries.

Blood alcohol and urine drug screens were completed for 1248 (85.0%) and 733 (49.9%) patients, respectively; 718 (48.9%) patients had both blood alcohol and urine drug tests completed (Fig. 1). Blood alcohol tests were completed a median of 2.3 h (IQR 1.8–3.2) post‐injury. Urine drug screens were completed a median of 8.8 h (IQR 4.7–18.1) post‐injury. Differences between patients with and without AOD tests are shown in Table 1. A lower percentage of patients with injuries caused by flames/scalds/contact burns and a higher percentage of trauma admissions had AOD testing completed. For alcohol testing, a higher percentage of interpersonal violence cases were tested. Relative to patients without urine drug screens, a higher percentage of patients with urine drug screens were male and had a hospital stay >1 day.

### Overall AOD detections

**Figure 2:**
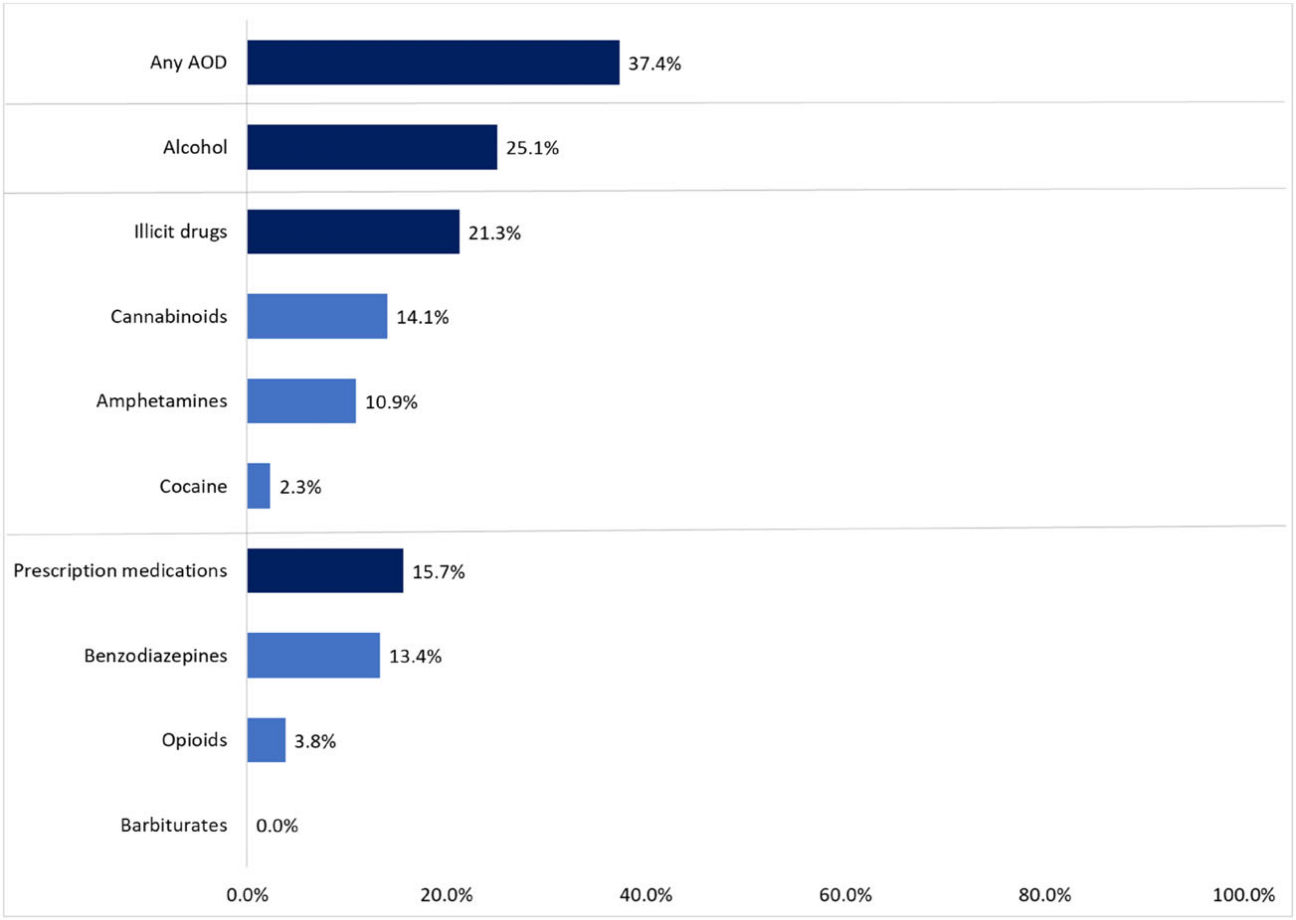
Prevalence of alcohol and/or other drug (AOD) detections in non‐transport injury events.

At least one substance was detected in 37.4% (*n* = 472) of the 1263 patients with a blood alcohol and/or urine drug screen completed (Table 2). The prevalence of AOD detections was high for both males (*n* = 368, 39.7%) and females (*n* = 104, 31.0%) and was consistently >40% in young and middle‐aged adults (Table 2). AOD detections were common in unintentional injury cases (*n* = 264, 28.6%) and even more prevalent in cases of intentional self‐harm (*n* = 50, 47.2%) and interpersonal violence (*n* = 123, 68.7%). Prevalence estimates were high across injury causes: high falls (*n* = 120, 27.6%), low falls (*n* = 119, 39.8%), flames/scalds/contact burns (*n* = 40, 43.0%), being struck by/colliding with an object/person (*n* = 89, 49.7%) and cutting/piercing objects (*n* = 92, 55.1%). The prevalence of specific AODs is shown in Figure 2 and described below.

**TABLE 2:** Proportion of non‐transport injury events with alcohol and/or other drug (AOD) detections†

	Any AOD (*n* = 1263)		Alcohol (*n* = 1248)		Illicit drugs‡ (*n* = 733)		Prescription drugs§ (*n* = 733)		Polysubstance use (*n* = 329)	
	Not detected	Detected	Not detected	Detected	Not detected	Detected	Not detected	Detected	One substance detected	Multiple substances detected
Total	791 (62.6)	472 (37.4)	935 (74.9)	313 (25.1)	577 (78.7)	156 (21.3)	618 (84.3)	115 (15.7)	210 (63.8)	119 (36.2)
Sex										
Male	560 (60.3)	368 (39.7)	665 (72.5)	252 (27.5)	420 (76.1)	132 (23.9)	475 (86.1)	77 (13.9)	158 (61.2)	100 (38.8)
Female	231 (69.0)	104 (31.0)	270 (81.6)	61 (18.4)	157 (86.7)	24 (13.3)	143 (79.0)	38 (21.0)	52 (73.2)	19 (26.8)
Age (years)										
18–24	73 (47.1)	82 (52.9)	90 (58.8)	63 (41.2)	50 (63.3)	29 (36.7)	72 (91.1)	7 (8.9)	31 (62.0)	19 (38.0)
25–34	100 (53.8)	86 (46.2)	121 (65.8)	63 (34.2)	72 (68.6)	33 (31.4)	94 (89.5)	11 (10.5)	32 (60.4)	21 (39.6)
35–44	89 (47.1)	100 (52.9)	124 (67.4)	60 (32.6)	78 (61.9)	48 (38.1)	97 (77.0)	29 (23.0)	41 (52.6)	37 (47.4)
45–54	111 (58.1)	80 (41.9)	140 (73.7)	50 (26.3)	88 (76.5)	27 (23.5)	90 (78.3)	25 (21.7)	33 (58.9)	23 (41.1)
55–64	125 (70.2)	53 (29.8)	140 (79.1)	37 (20.9)	–	≤15	96 (89.7)	11 (10.3)	–	≤15
≥65	293 (80.5)	71 (19.5)	320 (88.9)	40 (11.1)	–	≤15	169 (84.1)	32 (15.9)	–	≤15
Injury cause										
Low fall	180 (60.2)	119 (39.8)	220 (73.8)	78 (26.2)	127 (85.8)	21 (14.2)	113 (76.4)	35 (23.6)	59 (73.8)	21 (26.2)
High fall	315 (72.4)	120 (27.6)	353 (81.7)	79 (18.3)	247 (87.0)	37 (13.0)	257 (90.5)	27 (9.5)	65 (72.2)	25 (27.8)
Flames/scalds/contact burns	53 (57.0)	40 (43.0)	63 (73.3)	23 (26.7)	35 (70.0)	15 (30.0)	–	≤15	–	≤15
Cutting/piercing objects	75 (44.9)	92 (55.1)	108 (65.1)	58 (34.9)	55 (58.5)	39 (41.5)	69 (73.4)	25 (26.6)	32 (51.6)	30 (48.4)
Struck by/collided with an object/person	90 (50.3)	89 (49.7)	109 (60.9)	70 (39.1)	65 (63.1)	38 (36.9)	86 (83.5)	17 (16.5)	28 (46.7)	32 (53.3)
Other	78 (86.7)	12 (13.3)	82 (94.3)	5 (5.7)	48 (88.9)	6 (11.1)	–	≤15	–	≤15
Injury intent										
Unintentional	660 (71.4)	264 (28.6)	740 (81.1)	173 (18.9)	464 (86.2)	74 (13.8)	474 (88.1)	64 (11.9)	133 (70.0)	57 (30.0)
Intentional self‐harm	56 (52.8)	50 (47.2)	79 (76.7)	24 (23.3)	47 (72.3)	18 (27.7)	45 (69.2)	20 (30.8)	27 (69.2)	12 (30.8)
Interpersonal violence	56 (31.3)	123 (68.7)	97 (51.3)	92 (48.7)	43 (45.3)	52 (54.7)	70 (73.7)	25 (26.3)	36 (46.2)	42 (53.8)
Could not determine	19 (35.2)	35 (64.8)	29 (54.7)	24 (45.3)	23 (65.7)	12 (34.3)	29 (82.9)	6 (17.1)	14 (63.6)	8 (36.3)
High alcohol hours										
Yes	73 (38.2)	118 (61.8)	93 (48.9)	97 (51.1)	76 (73.8)	27 (26.2)	85 (82.5)	18 (17.5)	49 (66.2)	25 (33.8)
No	718 (67.0)	354 (32.0)	842 (79.6)	216 (20.4)	501 (79.5)	129 (20.5)	533 (84.6)	97 (15.4)	161 (63.1)	94 (36.9)

† Numerators indicate the number of positive detections. Denominators indicate the total number of patients in the specified cohort with a relevant test. Cells have been suppressed for variables where there was ≥1 cell with <5 patients.

‡ Illicit drugs included cannabinoids, amphetamine‐type substances and cocaine.

§ Prescription drugs included benzodiazepines and opioids. No barbiturates were detected.

### Alcohol

**Figure 3:**
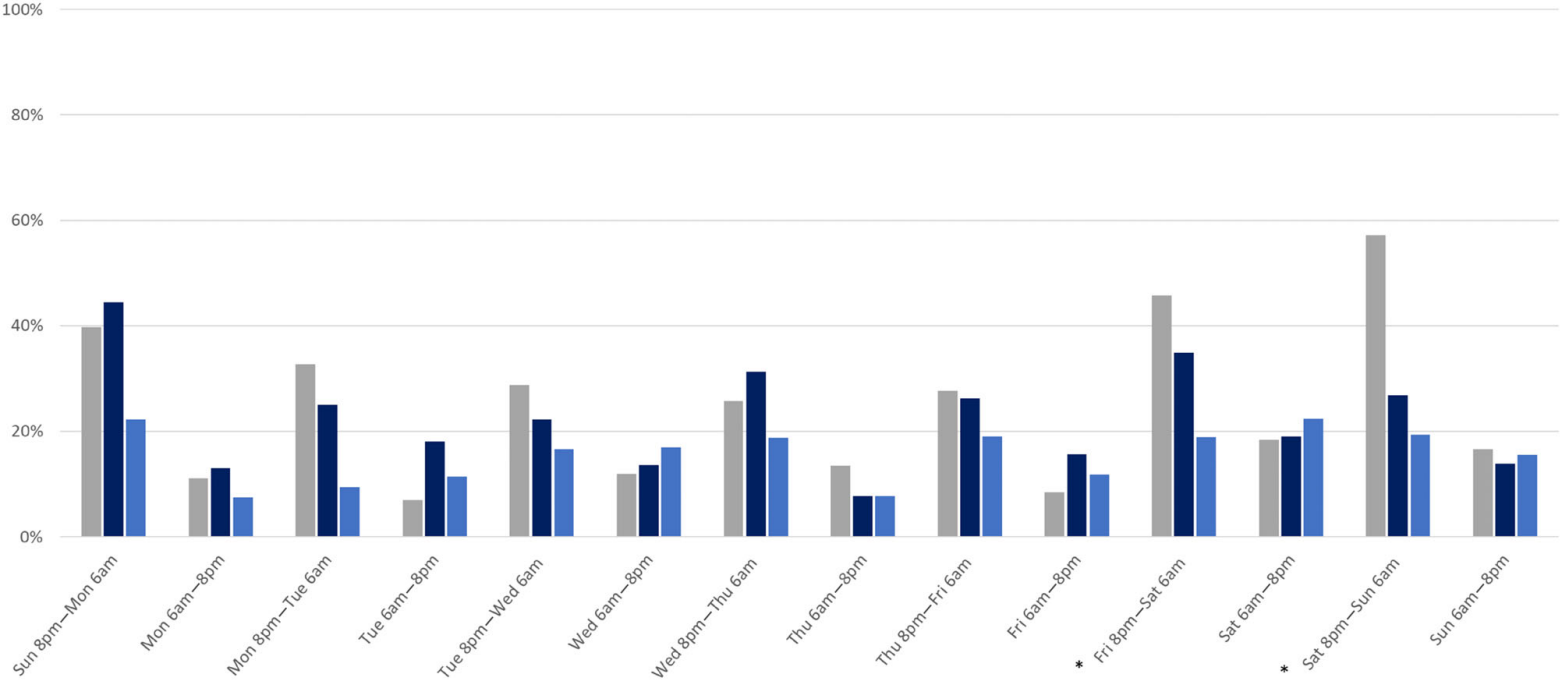
Prevalence of alcohol and/or other drug detections in non‐transport injury events, stratified by day and time. Asterisks (*) indicate high alcohol hours. See Table S2 for corresponding data. (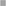), Alcohol; (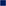), illicit drugs; (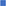), prescription drugs.

Alcohol was detected in 25.1% (*n* = 313/1248) of cases with a blood alcohol test. For cases where alcohol was detected, the median BAC was 0.20 g/100 mL (IQR 0.12–0.27; range 0.01–0.60). The prevalence of alcohol detections decreased as age increased (Table 2). Males had a higher alcohol prevalence compared to females (27.5% *vs* 18.4%). Alcohol was particularly common in cases of interpersonal violence (*n* = 92, 48.7%) and for injuries occurring on Friday and Saturday nights during high alcohol hours (*n* = 97/190, 51.5%; Fig. 3).

### Drugs other than alcohol

Drugs other than alcohol were detected in 31.1% (*n* = 228/733) of cases with a urine drug screen, including 156 (21.3%) detections for drugs considered to be illicit in Victoria during the study period (cannabinoids, amphetamine‐type substances, cocaine) and 115 (15.7%) prescription drug detections (opioids, benzodiazepines). No cannabinoid or amphetamine‐type substance detections were associated with prescribed drug use. There were no barbiturate detections.

#### Illicit drugs

Cannabinoids were the most commonly detected illicit drug (*n* = 103, 14.1%), followed by amphetamine‐type substances (*n* = 80, 10.9%) and cocaine (*n* = 17, 2.3%; Fig. 2). Illicit drug detections were more prevalent in males (*n* = 132/552, 23.9%) than females (*n* = 24/181, 13.3%). The age group with the highest prevalence was 35–44 years (*n* = 48/126, 38.1%), closely followed by 18–24 years (*n* = 29/79, 36.7%). Illicit drug detections were most common in injuries caused by cutting/piercing objects (*n* = 39/94, 41.5%), and in cases of interpersonal violence (*n* = 52/95, 54.7%) and intentional self‐harm (*n* = 18/65, 27.7%). Illicit drug detections were most prevalent in cases injured on Sunday nights (*n* = 20/45, 44.4%; Fig. 3). All patients identified as using prescribed amphetamine‐type substances tested positive for amphetamine‐type substances in urine drug screens (*n* < 5).

#### Prescription drugs

Benzodiazepines were detected in 13.4% (*n* = 98/733) of tested patients and opioids in 3.8% (*n* = 28/733). Most detections occurred in patients with pre‐existing prescriptions for these drugs (benzodiazepines: *n* = 62/98, 63.3%; opioids: *n* = 22/28, 78.6%). Females had a higher prevalence of prescription drug detections (*n* = 38/181, 21.0%) than males (*n* = 77/552, 13.9%). Prescription drug detections were common in injuries caused by cutting/piercing objects (*n* = 25, 26.6%) and low falls (*n* = 35, 23.6%), and in cases of intentional self‐harm (*n* = 20/65, 30.8%) and interpersonal violence (*n* = 25/95, 26.3%). Unlike alcohol and illicit drug detections, there was no clear peak regarding when prescription drug detections occurred. While the highest prevalence of prescription drug detections occurred between 6 am and 8 pm on Saturdays (22.4%), prevalence also ranged from 18.8% to 22.2% on Wednesday to Sunday nights (Fig. 3). Of the 677 patients identified as using opioids clinically (home, ambulance or in‐hospital use), 285 (42.1%) tested positive for opioids in urine drug screens. Meanwhile, 191 of the 294 patients identified as using benzodiazepines clinically (65.0%) tested positive for benzodiazepines in urine drug screens.

#### Polysubstance detections

At least one substance was detected in 45.8% (*n* = 329/718) of cases with both blood alcohol and urine drug screens. Polysubstance use was detected in 36.2% (*n* = 119/329) of those cases (Table 3). Two substances were detected in 83 cases, three in 26 cases and four in 10 cases. The detection of alcohol alone was most common at 32.2% (*n* = 106/329). Of the 228 cases who had a drug other than alcohol detected, multiple drugs other than alcohol were detected in 34.6% of cases (*n* = 79/228), including 17 cases where >2 drugs other than alcohol were detected.

**TABLE 3:** Prevalence of specific substance types in patients who had both blood alcohol and urine drug screens completed and had at least one substance detected (*n* = 329)†

	Alcohol (*n* = 175)	Cannabinoids (*n* = 100)	Amphetamine‐type substances (*n* = 79)	Benzodiazepines (*n* = 97)	Opiates (*n* = 27)	Cocaine (*n* = 17)
No other substance	106 (32.2%)	31 (9.4%)	20 (6.1%)	42 (12.8%)	8 (2.4%)	<5
Alcohol		33 (10.0%)	22 (6.7%)	29 (8.8%)	7 (2.1%)	13
Cannabinoids			37 (11.2%)	26 (7.9%)	6 (1.8%)	<5
Amphetamine‐type substances				24 (7.3%)	7 (2.1%)	<5
Benzodiazepines					11 (3.3%)	0
Opiates						<5
Cocaine						

† Some patients had detections for more than two substances. Percentages were calculated using the total number of patients who had both blood alcohol and urine drug data available and had at least one substance detected (*n* = 329) as the denominator. Cells have been suppressed for variables where there was a cell with <5 patients.

## Discussion

To our knowledge, this is the first study in Victoria to analyse data from routine AOD testing of biological samples for major trauma patients with non‐transport injury causes. Overall, AOD detections were prevalent in this sample. This highlights the need to address AOD use as a risk factor for all causes of injury, including non‐transport injury causes.

Police regulation has assisted with reducing drink driving‐related harms through interventions such as random breath testing and the enforcement of low blood alcohol limits while driving; however, these reductions have plateaued in recent decades.^1^ Meanwhile, AOD detections have remained high in non‐transport injury events. Prevalence estimates in the present study are similar to findings from a sample of South Australian trauma patients from 2007 where AODs were detected in 34% of fall‐related injuries, 49% of self‐harm injuries and 72% of interpersonal violence injuries.^11^ While comparisons across studies are limited by varying settings, objectives and testing protocols, current findings suggest that AODs may be more prevalent in non‐transport injury events relative to transport injury events. For example, both the prevalence and level of alcohol reported in the present study (25.1%, median BAC = 0.20 g/100 mL) are higher than that reported in a recent sample of hospitalised transport injury patients in Victoria based on blood samples (15.8%, median BAC = 0.15 g/100 mL).^2^ The prevalence of drugs other than alcohol in the present study (31.1%) was also substantially higher than the prevalence reported in a recent study of saliva samples from trauma patients injured in motor vehicle collisions in Victoria (13.2%).^12^

Taking a holistic, population‐based approach to prevention that acknowledges AOD use as a risk factor for all causes of injury has the potential to continue building on the reductions in harm achieved from targeted drink and drug‐driving countermeasures. This includes measures to reduce the widespread accessibility of alcohol (e.g. alcohol tax reforms, improved regulation of alcohol advertising, online alcohol sales and alcohol outlet densities),^13^, ^14^, ^15^ and the provision of community‐based AOD treatment and support services such as medically supervised injecting facilities, which have been shown to reduce exposure to violence in the people who attend them.^16^ Alcohol detections were common in cases injured on Friday and Saturday nights further supporting recommendations to consider policies that restrict late‐night alcohol trading hours for both on‐premise and off‐premise consumption, which have been associated with decreases in alcohol‐related harms.^17^ The high prevalence of AOD detections in this sample also reiterates the potential to use major trauma hospital admissions as an opportunity to deliver secondary interventions to mitigate repeated AOD‐related harms.^18^ Furthermore, it highlights the importance of preparing hospital staff and ensuring that EDs are equipped to safely manage injured patients who are intoxicated. Given the high prevalence of AOD detections in intentional self‐harm cases, this also aligns with recommendations from the Royal Commission into Victoria's Mental Health System regarding the need for EDs that can provide suitable AOD and mental health treatment for people in crisis.^19^

From a research perspective, there is need to better quantify the risk of various non‐transport injury causes associated with AOD use, and to improve our understanding of the interaction between AOD use, risk‐taking behaviour and risk of injury. This will help inform future prevention strategies that are tailored to specific injury causes. For example, research on the role of polypharmacy in low falls has highlighted opportunities for prevention in older populations.^20^

A strength of the present study was the use of biological samples, which are a more sensitive measure of AOD involvement in injury events compared to administrative coding.^21^ However, there are some limitations that may have resulted in the underestimation of the prevalence of AODs at the time of injury. Time delays between the injury event and hospital presentation likely allowed patient AOD levels to decline, possibly to undetectable levels. Furthermore, urine drug screens were limited to six drug classes. While drugs such as ketamine and antipsychotics were not commonly detected in injured patients from previous studies, others such as antidepressants are relatively prevalent.^2^, ^22^ Meanwhile, other limitations may have resulted in the overestimation of AOD detections. For example, half of the current sample was missing urine drug screen data and it is possible that AOD testing was more likely to be completed for patients who were clinically intoxicated. While urine drug screens were routinely ordered for patients who met trauma call‐out criteria, urine samples can be difficult to obtain in clinical settings, especially for patients with short hospital stays. Furthermore, urine samples are associated with a longer window of detection for substances relative to blood samples, meaning that we may have detected drugs that were used in the days leading up to injury that were unrelated to the injury event.^23^ This may, at least in part, have contributed to the high prevalence of illicit drug detections on Sunday nights, as well as the relatively large prevalence estimates observed on Friday and Saturday nights. Blood samples may provide a better alternative to urine samples as the detection window is tighter and they are already obtained via routine venepuncture at the time of admission for a high proportion of trauma patients.

AOD detections do not necessarily infer that AODs contributed to the injury event. Patients who engage in chronic AOD use can develop tolerance to the intoxicating effects of AODs. This is supported by the detection of BACs as high as 0.60 g/100 mL in the present study, which are typically considered to be potentially fatal.^24^ Additionally, patients may have used AODs after the injury event, but before presenting to hospital. In the present study, we accounted for post‐injury clinical drug administration using a conservative approach. Prescription drug detections were recoded as ‘not detected’ if the patient had been given those drugs by clinical staff post‐injury; however, some patients may also have used these drugs before the injury event. Additionally, it is possible that some medications may not have been listed in patient medical records (e.g. over‐the‐counter preparations that are not reported by the patient). Data on the level of drugs detected were not available. Therefore, it is unknown whether patients were using prescription drugs as recommended. The present study only included AOD measurements for patients who were injured. Some injury events may have involved other intoxicated individuals (e.g. perpetrators of violence).

As a single‐site study, findings may not generalise to other sites. Larger, population‐level datasets would enable more granular analyses of drugs other than alcohol in injury events. The generalisability of findings to trauma patients with less serious injuries is limited as AOD testing was restricted to cases that met trauma call‐out criteria.

## Conclusion

AOD detections were prevalent in non‐transport injury events. While AOD detections were common in most injury cause and intent groups, prevalence was particularly high in cases of intentional self‐harm and interpersonal violence. Although routine AOD screening was completed for clinical purposes in this setting, there were clear benefits for the surveillance of AODs in injury events. Our study highlights how surveillance systems can be used to help target interventions and resources. Findings demonstrate the need to establish whole‐of‐population surveillance systems that can be used to inform the implementation of prevention strategies that address AOD use as a risk factor for all causes of injury.

## Supporting information


**Table S1.** Lower detection limits for AOD testing.
**Table S2.** Prevalence of alcohol and other drug detections in non‐transport injury patients, stratified by day and time.

## Data Availability

The data that support the findings of this study are not publicly available due to privacy and ethical restrictions.
