# Supplementary material for: Prevalence of alcohol and other drug detections in non‐transport injury events
Source: Emerg Med Australas. 2023 Sep 17;36(1):78–87. doi: 10.1111/1742-6723.14312 (PMC10952644; doi:10.1111/1742-6723.14312)
Supplement: Supplementary file 1 — Table S1. Lower detection limits for AOD testing. Table S2. Prevalence of alcohol and other drug detections in non‐transport injury patients, stratified by day and time. [file EMM-36-78-s001.docx]

# Supplementary Material

## Table S1. Lower detection limits for AOD testing.

| **AOD type** | **Sample type** | **Lower limit** |
| --- | --- | --- |
| Alcohol | Blood | 2mmol/L |
| Amphetamine-type substances | Urine | 300μg/L |
| Barbiturates | Urine | 200μg/L |
| Benzodiazepines | Urine | 200μg/L |
| Cannabinoids | Urine | 50μg/L |
| Cocaine | Urine | 300μg/L |
| Opioids | Urine | 300μg/L |

## Table S2. Prevalence of alcohol and other drug detections in non-transport injury patients, stratified by day and time.^a^

| Time | Alcohol | | Illicit drugs | | Prescription drugs | |
| --- | --- | --- | --- | --- | --- | --- |
|  | Not detected  n (%) | Detected  n (%) | Not detected  n (%) | Detected  n (%) | Not detected  n (%) | Detected  n (%) |
| Sun 8pm - Mon 6am | 44 (60.3) | 29 (39.7) | 25 (55.6) | 20 (44.4) | 35 (77.8) | 10 (22.2) |
| Mon 6am - 8pm | 80 (88.9) | 10 (11.1) | - | <8 | - | <8 |
| Mon 8pm - Tue 6am | 39 (67.2) | 19 (32.8) | 24 (75.0) | 8 (25 .0) | - | <8 |
| Tue 6am - 8pm | 94 (93.1) | 7 (6.9) | 50 (82.0) | 11 (18.0) | - | <8 |
| Tue 8pm - Wed 6am | 42 (71.2) | 17 (28.8) | 28 (77.8) | 8 (22.2) | - | <8 |
| Wed 6am - 8pm | 81 (88) | 11 (12) | 51 (86.4) | 8 (13.6) | 49 (83.1) | 10 (16.9) |
| Wed 8pm - Thu 6am | 49 (74.2) | 17 (25.8) | 33 (68.7) | 15 (31.3) | 39 (81.2) | 9 (18.8) |
| Thu 6am - 8pm | 77 (86.5) | 12 (13.5) | - | <8 | - | <8 |
| Thu 8pm - Fri 6am | 55 (72.4) | 21 (27.6) | 31 (73.8) | 11 (26.2) | 34 (81.0) | 8 (19.0) |
| Fri 6am - 8pm | 87 (91.6) | 8 (8.4) | 43 (84.3) | 8 (15.7) | - | <8 |
| Fri 8pm - Sat 6am^b^ | 57 (54.3) | 48 (45.7) | 28 (65.1) | 15 (34.9) | 43 (81.1) | 10 (18.9) |
| Sat 6am - 8pm | 80 (81.6) | 18 (18.4) | 47 (81.0) | 11 (19.0) | 45 (77.6) | 13 (22.4) |
| Sat 8pm - Sun 6am^b^ | 51 (42.9) | 68 (57.1) | 49 (73.1) | 18 (26.9) | 54 (80.6) | 13 (19.4) |
| Sun 6am - 8pm | 75 (83.3) | 15 (16.7) | 50 (86.2) | 8 (13.8) | 49 (84.5) | 9 (15.5) |

^a^Cells have been suppressed as there was ≥1 cell with <5 detections. ^b^Included in high alcohol hours.
